# Supplementary material for: Causal Relationship between Aging and Anorexia Nervosa: A White-Matter-Microstructure-Mediated Mendelian Randomization Analysis
Source: Biomedicines. 2024 Aug 16;12(8):1874. doi: 10.3390/biomedicines12081874 (PMC11351342; doi:10.3390/biomedicines12081874)
Supplement: Supplementary file 1 [file biomedicines-12-01874-s001.zip › Supplementary Figure/Figure S8/Leaveoneout plot of MR analysis for TL to ubm-b-1619.pdf]

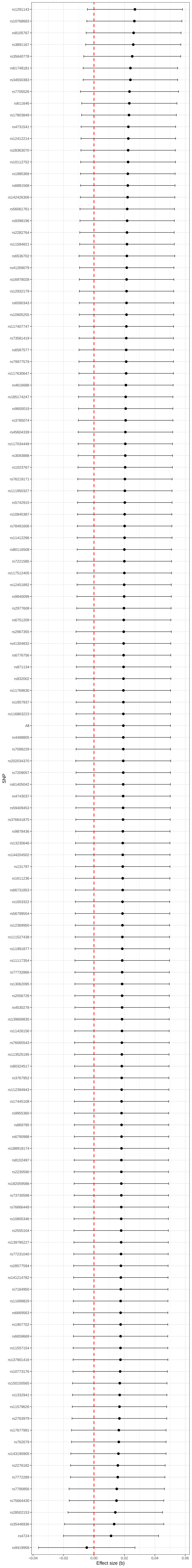
rs1291143
rs10768683
rs8105767
rs3891167
rs35640778
rs61748181
rs34550383
rs7705526
rs611646
rs17803849
rs4731541
rs12412214
rs28363070
rs10112752
rs1985369
rs6881568
rs142426306
rs56061761
rs9398196
rs2282764
rs11584821
rs6536702
rs41269079
rs16978028
rs12932179
rs6590343
rs10905255
rs117407747
rs73581419
rs6587577
rs79977579
rs117630647
rs4616688
rs185174247
rs9600019
rs3785074
rs45604339
rs117034449
rs3093888
rs1023767
rs76219171
rs111950327
rs5742915
rs10845387
rs78491606
rs11412296
rs80116508
rs7221585
rs117512405
rs12451892
rs9940099
rs2977608
rs6751209
rs2967355
rs41304832
rs6776756
rs871134
rs932002
rs11769630
rs1957937
rs116863223
All
rs4498805
rs7099229
rs202034370
rs7209057
rs61405042
rs4743037
rs59409453
rs376641875
rs9878436
rs13230646
rs144204502
rs131797
rs1611236
rs66731853
rs1003322
rs56799554
rs12369950
rs111527438
rs11991877
rs11117354
rs77732866
rs13062095
rs2056726
rs4530278
rs139669835
rs11426156
rs76065543
rs113525195
rs80324517
rs3767952
rs112394943
rs17445108
rs9955360
rs869785
rs6790988
rs188918174
rs8102497
rs2230590
rs182059586
rs73730598
rs76666449
rs10805346
rs2555104
rs139795227
rs77231040
rs28577594
rs141214782
rs7164950
rs11699829
rs6669563
rs1907702
rs6659669
rs11557154
rs137901416
rs10773176
rs150150565
rs1332941
rs11579626
rs2763979
rs17677991
rs762679
rs143190905
rs2276182
rs7772289
rs7790856
rs75664430
rs28502153
rs35446936
rs4724
rs9419958
SNP
-0.04
-0.02
0.00
0.02
0.04
0.06
Effect size (b)
